# Attenuation Effects of Alpha-Pinene Inhalation on Mice with Dizocilpine-Induced Psychiatric-Like Behaviour

**DOI:** 10.1155/2019/2745453

**Published:** 2019-07-30

**Authors:** Hiroshi Ueno, Atsumi Shimada, Shunsuke Suemitsu, Shinji Murakami, Naoya Kitamura, Kenta Wani, Yosuke Matsumoto, Motoi Okamoto, Takeshi Ishihara

**Affiliations:** ^1^Department of Medical Technology, Kawasaki University of Medical Welfare, Okayama, 701-0193, Japan; ^2^Division of Food and Nutrition, Nakamura Gakuen University Junior College, Fukuoka, 814-0198, Japan; ^3^Department of Psychiatry, Kawasaki Medical School, Kurashiki, 701-0192, Japan; ^4^Department of Neuropsychiatry, Graduate School of Medicine, Dentistry and Pharmaceutical Sciences, Okayama University, Okayama, 700-8558, Japan; ^5^Department of Medical Technology, Graduate School of Health Sciences, Okayama University, Okayama, 700-8558, Japan

## Abstract

*α*-Pinene, an organic terpene compound found in coniferous trees, is used as a safe food additive and is contained in many essential oils. Moreover, some studies have shown that *α*-pinene suppresses neuronal activity. In this study, we investigated whether inhalation of *α*-pinene suppresses dizocilpine (MK-801-) induced schizophrenia-like behavioural abnormalities in mice. Mice inhaled *α*-pinene 1 h before the first MK-801 injection. Thirty minutes after MK-801 injection, the open field, spontaneous locomotor activity, elevated plus maze, Y-maze, tail suspension, hot plate, and grip strength tests were conducted as behavioural experiments. Inhalation of *α*-pinene suppressed the activity of mice in the spontaneous locomotor activity test and although it did not suppress the MK-801-induced increased locomotor activity in the open field test, it remarkably decreased the time that the mice remained in the central area. Inhalation of *α*-pinene suppressed the MK-801-induced increased total distance travelled in the Y-maze test, whereas it did not alter the MK-801-induced reduced threshold of antinociception in the hot plate test. In the tail suspension and grip strength tests, there was no effect on mouse behaviour by administration of MK-801 and inhalation of *α*-pinene. These results suggest that *α*-pinene acts to reduce MK-801-induced behavioural abnormalities resembling those seen in neuropsychiatric disorders. Therefore, both medicinal plants and essential oils containing *α*-pinene may have potential for therapeutic treatment of schizophrenia.

## 1. Introduction

Schizophrenia is a chronic and degenerative disease, with an overall lifetime risk of 1%. Despite its high prevalence, the pathogenesis of schizophrenia is not clear [1, 2]. Current antipsychotics are generally successful in treating positive symptoms (e.g., hallucinations and delusions) but have limited effect on the reduction of negative and cognitive symptoms and have side effects [3–6]. Therefore, there is a need for new therapeutic agents with fewer side effects. The side effects accompanying the use of synthetic drugs, together with their acquisition cost and supply shortage, have recently increased people's interest in using medicinal plants. Aromatherapy, an alternative type of medicine which uses medicinal plants, is widely used for the management of chronic pain, depression, anxiety, insomnia, and stress-related disorders [7, 8]. However, the scientific basis underlying the mechanism of action of many medicinal plants and essential oils remains unclear [8, 9]. Therefore, the need to clarify the scientific mechanism of medicinal plants considered to have a physiological effect is rapidly becoming more urgent [10]. Additionally, these medicinal plants are also an important source of new chemicals with potential therapeutic effects.

*α*-Pinene is an organic terpene compound, which is contained in the oil of coniferous trees, and is the major monoterpene in pine trees [11]. *α*-Pinene is widely used as a food-flavouring ingredient [12, 13] and has been approved as a safe food additive by the U.S. Food and Drug Administration [14]. In addition, *α*-pinene is also contained in essential oils such as in rosemary (*Rosmarinus officinalis*) oil,* Eucalyptus* oil, camphor,* Bupleurum fruiticescens*,* Psidium*, and* Opuntia humifusa*. Indeed, *α*-pinene is considered to have a physiological effect on humans [15, 16], and essential oils containing *α*-pinene have been used to treat several diseases [17]. However, in humans, odours may lead to behavioural and cognitive changes, so careful interpretation is necessary. Inhalation of essential oils transfers signals from the olfactory system to the brain, and the brain regulates anxiety, depression, and mood disorders by secreting neurotransmitters such as serotonin and dopamine [7]. Therefore, various plant-derived essential oils have traditionally been used to treat psychiatric disorders such as depression, anxiety neurosis, attention-deficit hyperactivity disorder, and bipolar disorder. In addition, it has been reported that *α*-pinene also has anti-inflammatory [18, 19], antidepressant [20], anticonvulsant [21], antioxidant [22], antitumoral [23], and antinociceptive effects [24]. In recent years, it has been reported that inhalation of *α*-pinene has anxiolytic effects on mice during the elevated plus maze test [25]. Inhalation of *α*-pinene also leads to the accumulation of *α*-pinene in the brain. Furthermore, it has been shown that inhalation of *α*-pinene significantly increases rapid eye movement in rats during sleep [26]. Although *α*-pinene reportedly acts on rodents' nerves by inhalation, it has not been investigated how *α*-pinene affects psychiatric-like behavioural abnormalities. Considering that *α*-pinene acts on the nervous system, *α*-pinene may be a potential therapeutic agent for psychoneurotic diseases including schizophrenia.

N-methyl-D-aspartate (NMDA) receptor antagonists such as dizocilpine (MK-801) are widely used for animal models of schizophrenia [27–30]. Administration of MK-801 alters the glutamatergic system in animal models and causes positive symptoms, negative symptoms, and cognitive impairment, similar to those seen in schizophrenia [31–35]. Many studies have reported the involvement of glutamatergic neurotransmission dysfunction via NMDA receptors in the pathophysiology of schizophrenia [36–40]. It is well established that the NMDA receptor plays an important role in the pathogenesis and pharmacological treatment of schizophrenia [33–35, 41]. Thus, when a novel compound relieves MK-801-induced behavioural abnormalities, a range of tests can be widely used to evaluate its preclinical utility as a potential antipsychotic [42–44].

This study aimed to evaluate whether inhalation of *α*-pinene suppresses schizophrenia-like behaviour abnormalities induced by MK-801. We investigated whether MK-801-induced abnormal behaviours would be suppressed by preexposure to *α*-pinene through a series of behavioural experiments.

## 2. Experimental Procedures

### 2.1. Animals

We used 15-week-old male mice (C57BL/6) for the experiments. We purchased the animals from Charles River Laboratories (Kanagawa, Japan) and housed them in cages with food and water provided* ad libitum* under a 12 h light/dark cycle at 23–26°C. We made every effort to minimize the number of animals used and their suffering. These experiments complied with the U.S. National Institutes of Health (NIH) Guide for the Care and Use of Laboratory Animals (NIH Publication No. 80-23, revised in 1996) and were approved by the Committee for Animal Experiments at Kawasaki Medical School Advanced Research Center.

### 2.2. Drug Administration

(+)-MK-801 (dizocilpine hydrogen maleate; 130-17381, FUJIFILM Wako Pure Chemical Corporation, Osaka, Japan) was diluted in saline at concentrations of 0.1 mg/mL and administered intraperitoneally (i.p.) in a volume of 0.2 mg/kg. This dose was selected on the basis of previous studies showing a schizophrenia-related deficient effect of MK-801 at 0.2 mg/kg in mice [45–47].

### 2.3. Inhalation of *α*-Pinene

*α*-Pinene was acquired from FUJIFILM Wako Pure Chemical Corporation (169-21242). Additionally, saline was used as a control. The inhalation apparatus was the same as that used in a previous study [48]. Inhalation of the odour was carried out in a sealed container. A piece of absorbent cotton (4 × 4 cm) impregnated with 2 mL of *α*-pinene was placed in a stainless-steel container (60 × 60 × 35 mm) capped by a lid with holes. The mice were unable to lick or touch the cotton. The stainless-steel container was placed in a new breeding cage (235 × 325 × 170 mm) surrounded by two larger cages (292 × 440 × 200 mm). Approximately 20 min after the cotton placement, the mice were placed in the internal cage for 30 min. Control group mice were placed in the same container without the *α*-pinene. After 30 min, the mice received either a dose of MK-801 or saline. The test was started 30 min after MK-801 treatment.

### 2.4. Behavioural Tests

All behavioural experiments were performed during the light phase (9:00–16:00). We tested mice in a random order. After testing, the apparatus was cleaned with 70% ethanol and 80 ppm super hypochlorous water to prevent any bias due to olfactory cues [49]. Animals were randomly selected and divided, according to a table of random numbers, into four groups: a control (inhaled saline, administered saline), MK-801 group (inhaled saline, administered MK-801), MK-801 + *α*-pinene group (inhaled *α*-pinene, administered MK-801), and *α*-pinene group ((inhaled *α*-pinene, administered saline). Each animal was subjected to the open field test only once (n = 8 animals per group). Next, the animals were randomly divided into three groups: a control group, MK-801 group, and MK-801 + *α*-pinene group. Each animal was subjected to the locomotor activity test, elevated plus maze test, Y-maze test, hot plate test, neurological screening, and tail suspension test only once (n = 10 animals per group).

### 2.5. Locomotor Activity Test

To assess whether exposure to *α*-pinene induced motor impairment or not, we examined locomotor activity under odour exposure. For measurements of locomotor activity, the mice were acclimated to the single housing environment (235 mm × 325 mm × 170 mm) for 3 h. Locomotor activity data were measured using a photobeam activity system (ACTIMO-100; BRC Co., Nagoya, Aichi, Japan). Sensors were located every 2 cm along the floor of the enclosure. Activity counts were expressed as the number of ambulations recorded at 10-min intervals. Mice were placed into photobeam activity arenas for 60 min before those in the MK-801 or saline groups were administered an injection [50]. Locomotion was measured for 3 h. “Control” group was treated with dH_2_O (inhalation) during the test period. “MK-801” group was treated with MK-801 (i.p.) and dH_2_O (inhalation) during the test period. “MK-801 + *α*-pinene” group was treated with MK-801 (i.p.) and *α*-pinene (inhalation) during the test period.

### 2.6. Open Field Test

In the open field test, each mouse was placed at the centre of the apparatus, consisting of a square area surrounded by high walls (40 × 40 × 40 cm). The total distance travelled (m) and the time spent in the central area (s) were recorded [51]. The central area was defined as the 20 × 20-cm area located at the centre of the field. The test chamber was illuminated at 100 lux. Data were collected over a 30-min period. Data analysis was performed automatically using video tracking software (ANY-MAZE, Stoelting Co., Wood Dale, IL, USA).

### 2.7. Elevated Plus Maze Test

The apparatus consisted of two open arms (8 × 25 cm) and two closed arms of the same size with 30 cm-high transparent walls, similar to previous studies [52, 53]. The arms were made of white plastic plates and elevated to a height of 40 cm above the floor. Arms of the same type were located opposite each other. Each mouse was placed at the central square of the maze, facing one of the closed arms, and was allowed to move freely between the two arms for 10 min. The number of arm entries, distance travelled (m), and time (s) spent in the open arms were video recorded and analysed using the ANY-MAZE software.

### 2.8. Y-Maze Test

Spatial working memory was measured using a Y-maze apparatus (arm length: 40 cm, arm bottom width: 3 cm, arm upper width: 10 cm, wall height: 12 cm). The protocol was similar to that used in a previous study [54]. Mice were placed at the centre of the Y-maze field. Visual cues were placed around the maze in the testing room and were constant throughout the testing sessions. Mice were examined with no prior learning. The number of entries and alterations was recorded and analysed automatically using the ANY-MAZE software. Data were collected for 10 min.

### 2.9. Hot Plate Test

The hot plate test was used to evaluate the nociception or sensitivity to a painful stimulus [55]. It consisted of an electrically heated surface and an open Plexiglass box (20 × 20 × 30 cm) to contain the animals. Mice were placed on a hot plate at 55.0 ± 0.3°C, and the latency to the first hind-paw response was recorded. The hind-paw responses counted foot shakes or paw licks. A latency period of 30 s was defined as complete analgesia and used as the cut-off time to prevent tissue injuries.

### 2.10. Neurological Screening

Neuromuscular strength was examined using the grip strength test according to a previous study [56]. A grip strength metre was used to assess forelimb grip strength. Mice were lifted and held by the tail such that their forepaws could grasp a wire grid; they were then pulled backward gently until they released the grid. The peak force applied by the forelimbs was recorded in Newtons (cN).

### 2.11. Tail Suspension Test

Each mouse was suspended by the tail at 60 cm above the floor, in a white plastic chamber, using adhesive tape placed <1 cm from the tip of the tail. Mouse behaviour was recorded for 6 min. Images were captured via a video camera, and immobility time was measured [51]. In this test, the ‘immobile period' was defined as the period when the animals stopped struggling for ≥ 1 s. Data acquisition and analysis were performed automatically using the ANY-MAZE software.

### 2.12. Statistical Analyses of Behavioural Test Results

Data were analysed using one-way analysis of variance (ANOVA) followed by Tukey's test, two-way repeated measures ANOVA followed by Fisher's LSD test, Student's t-test, or paired t-test. A p-value of < 0.05 was regarded as statistically significant. Data are presented as box plots.

## 3. Results

### 3.1. Effect of *α*-Pinene on MK-801-Induced Abnormal Behaviour in the Locomotor Activity Test

**Figure 1:**
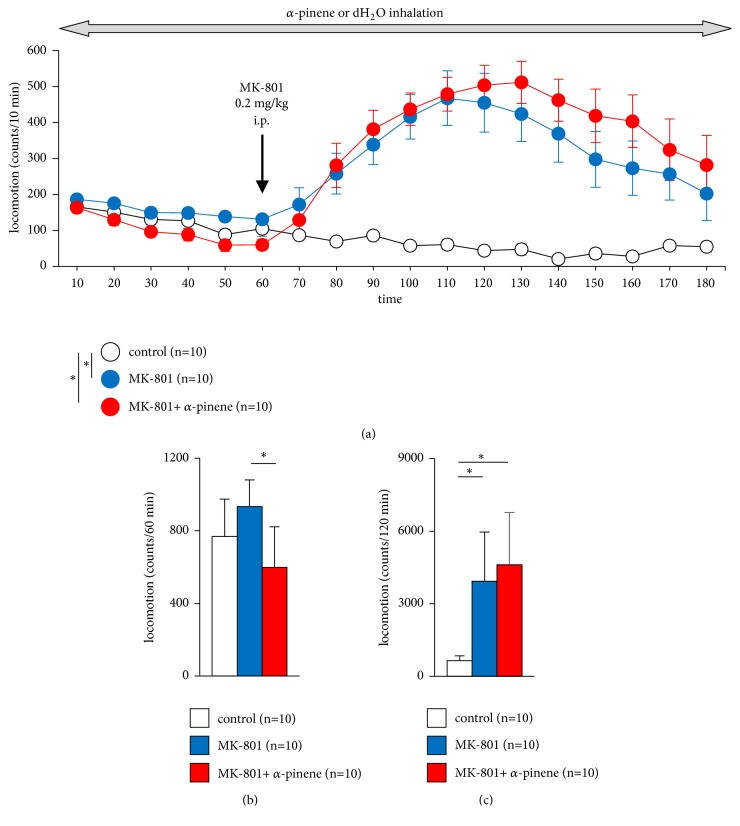
Effect of *α*-pinene on MK-801-induced hyperlocomotion in the locomotor activity test. Results of the locomotor activity test in the three groups. Spontaneous locomotor activity in each 10-min period (a). After 60 min, animals were injected with MK-801 or saline, and locomotor activity was assessed for 180 min. Total beam breaks for 60 min before injection of MK-801 or saline (b). Total beam breaks for 120 min after injection of MK-801 or saline (c). Data are presented as the mean ± SEM (a), or box plots (b, c). *∗*, significant difference among groups (p < 0.05). The p values were calculated using two-way repeated measures analysis of variance in (a) and Student's t-test in ((b), (c)).

First, we tested whether *α*-pinene affects locomotor activity. Mice were pretreated with *α*-pinene before administration of MK-801. Pretreatment with *α*-pinene reduced basal activity (Figure 1(a),* F*_17,459_ = 10.07, p < 0.001; control vs. MK-801, p = 0.001; control vs. MK-801 + *α*-pinene, p < 0.001, MK-801 vs. MK-801 + *α*-pinene, p = 1.0, B,* F*_2,29_ = 7.348, p = 0.003; control vs. MK-801, p = 0.164; control vs. MK-801 + *α*-pinene, p = 0.143, MK-801 vs. MK-801 + *α*-pinene, p = 0.002). MK-801, an NMDA receptor antagonist, increases locomotor activity [57]. As shown in Figure 1(a), the injection with MK-801 resulted in a robust increase in locomotor activity, which lasted for a further 120 min (Figure 1(a)). While the *α*-pinene pretreatment had a clear effect on basal activity, there was no effect on the MK-801-induced locomotor activity (Figures 1(a) and 1(c),* F*_2,29_ = 15.134, p < 0.001; control vs. MK-801, p = 0.001; control vs. MK-801 + *α*-pinene, p < 0.001, MK-801 vs. MK-801 + *α*-pinene, p = 0.654).

### 3.2. Effect of *α*-Pinene on MK-801-Induced Abnormal Behaviour in the Open Field Test

**Figure 2:**
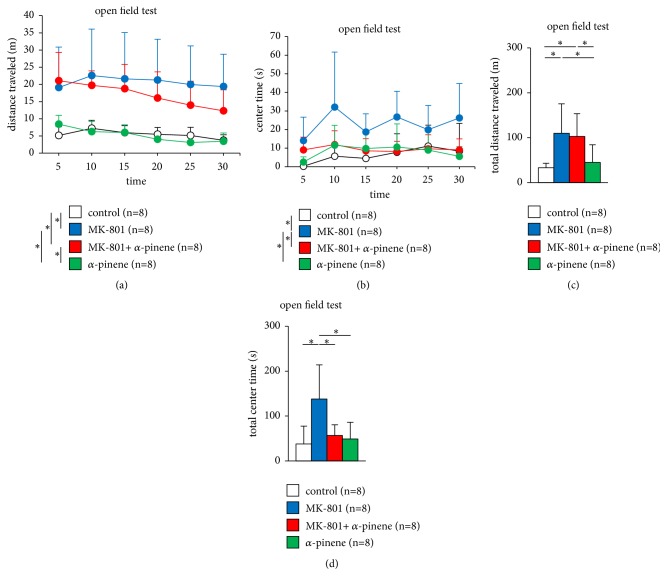
Effect of *α*-pinene on MK-801-induced hyperlocomotion in the open field test. Graphs showing the distance travelled (a) and time spent in the central area (b) in each 5 min-period of the open field test. Graphs showing the total distance travelled (c) and total time spent in the central area (d) in the open field test. All data are presented as box plots. *∗*, significant difference among groups (p < 0.05). The p-values were calculated using two-way repeated measures analysis of variance in (a, b) and one-way analysis of variance in ((c), (d)).

In the open field test, we observed no significant difference in the total distance travelled between mice exposed to only *α*-pinene and control mice (Figure 2(a),* F*_15,140_ = 2.275, p = 0.007; control vs. MK-801, p < 0.001; control vs. MK-801 + *α*-pinene, p = 0.008, control vs. *α*-pinene, p = 1.0, MK-801 vs. MK-801 + *α*-pinene, p = 1.0, MK-801 vs. *α*-pinene, p < 0.001, MK-801 + *α*-pinene vs. *α*-pinene, p = 0.007, Figure 2(c),* F*_3,31_ = 5.762, p = 0.003; control vs. MK-801, p = 0.012; control vs. MK-801 + *α*-pinene, p = 0.026, control vs. *α*-pinene, p = 0.952, MK-801 vs. MK-801 + *α*-pinene, p = 0.99, MK-801 vs. *α*-pinene, p = 0.043, MK-801 + *α*-pinene vs. *α*-pinene, p = 0.083). No differences were observed in the time spent in the central area between mice exposed to only *α*-pinene and control mice (Figure 2(b),* F*_15,140_ = 1.133, p = 0.333; control vs. MK-801, p = 0.002; control vs. MK-801 + *α*-pinene, p = 1.0, control vs. *α*-pinene, p = 1.0, MK-801 vs. MK-801 + *α*-pinene, p = 0.013, MK-801 vs. *α*-pinene, p = 0.006, MK-801 + *α*-pinene vs. *α*-pinene, p = 1.0, Figure 2(d),* F*_3,31_ = 7.186, p = 0.001; control vs. MK-801, p = 0.002; control vs. MK-801 + *α*-pinene, p = 0.864, control vs. *α*-pinene, p = 0.966, MK-801 vs. MK-801 + *α*-pinene, p = 0.011, MK-801 vs. *α*-pinene, p = 0.005, MK-801 + *α*-pinene vs. *α*-pinene, p = 0.989).

Administration of MK-801 markedly increased locomotor activity in mice (Figures 2(a) and 2(c)). Similarly, administration of MK-801 markedly increased locomotor activity in mice preexposed to *α*-pinene (Figures 2(a) and 2(c)). We observed no significant difference in the MK-801-induced hyperlocomotion between mice preexposed to *α*-pinene and those exposed to saline.

The time spent in the central area was significantly higher in mice administered MK-801 than in control mice (Figures 2(b) and 2(d)). Pretreatment with *α*-pinene significantly attenuated the MK-801-induced longer time spent in the central area (Figures 2(b) and 2(d)).

### 3.3. Effect of *α*-Pinene on MK-801-Induced Abnormal Behaviour in the Elevated Plus Maze Test

**Figure 3:**
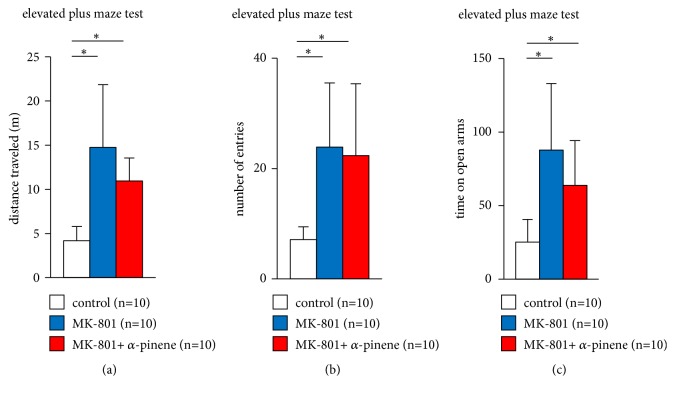
Effect of *α*-pinene on MK-801-induced abnormal behaviour in the elevated plus maze test. Graphs showing the total distance travelled (a), the number of open arm entries (b), and the time spent in the open arms (c) in the elevated plus maze test. All data are presented as box plots. *∗*, significant difference among groups (p < 0.05). The p-values were calculated using one-way analysis of variance in ((a)–(c)).

In the elevated plus maze test, we evaluated anxiety-like behaviour in mice administered MK-801 after preexposure to *α*-pinene. Administration of MK-801 markedly increased the total distance travelled in mice preexposed to saline or *α*-pinene (Figure 3(a),* F*_2,28_ = 14.157, p < 0.001; control vs. MK-801, p < 0.001; control vs. MK-801 + *α*-pinene, p = 0.007, MK-801 vs. MK-801 + *α*-pinene, p = 0.18).

Mice administered MK-801 had a significantly higher number of entries to open arms than did the control mice (Figure 3(b),* F*_2,28_ = 8.342, p = 0.002; control vs. MK-801, p = 0.003; control vs. MK-801 + *α*-pinene, p = 0.007, MK-801 vs. MK-801 + *α*-pinene, p = 0.942). Similarly, mice administered MK-801 spent markedly increased time in the open arms than did the control mice (Figure 3(c),* F*_2,28_ = 9.183, p = 0.001; control vs. MK-801, p = 0.001; control vs. MK-801 + *α*-pinene, p = 0.039, MK-801 vs. MK-801 + *α*-pinene, p = 0.269).

No differences were observed in the total distance travelled, the number of total entries into open arms, and the time spent in open arms between mice administered MK-801 exposed to *α*-pinene or exposed to saline.

### 3.4. Effect of *α*-Pinene on MK-801-Induced Abnormal Behaviour in the Y-Maze Test

**Figure 4:**
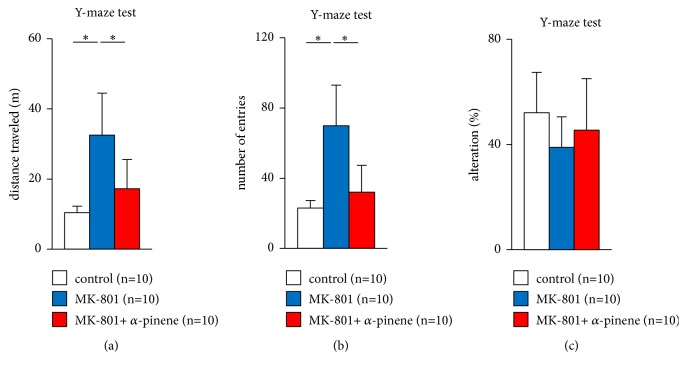
Effect of *α*-pinene on MK-801 induced cognitive deficits in the Y-maze test. Graphs showing the total distance travelled (a), total number of arm entries (b), and percentage of alterations (c). All data are presented as box plots. *∗*, significant difference among groups (p < 0.05). The p-values were calculated using the one-way analysis of variance in ((a)–(c)).

We examined the effect of *α*-pinene on the short-term spatial working memory of mice administered MK-801 by monitoring spontaneous alteration behaviour in the Y-maze test. Administration of MK-801 markedly increased locomotor activity in mice (Figure 4(a),* F*_2,28_ = 13.284, p < 0.001; control vs. MK-801, p < 0.001; control vs. MK-801 + *α*-pinene, p = 0.191, MK-801 vs. MK-801 + *α*-pinene, p = 0.004). Similarly, administration of MK-801 markedly increased the number of arm entries (Figure 4(b),* F*_2,28_ = 17.62, p < 0.001; control vs. MK-801, p < 0.001; control vs. MK-801 + *α*-pinene, p = 0.435, MK-801 vs. MK-801 + *α*-pinene, p < 0.001). There were no significant differences between control mice and mice administered MK-801 preexposed to *α*-pinene in the total distance travelled (Figure 4(a)) and the number of arm entries (Figure 4(b)). No differences were observed in the alteration percentage among the three groups (Figure 4(c),* F*_2,28_ = 1.086, p = 0.356; control vs. MK-801, p = 0.326; control vs. MK-801 + *α*-pinene, p = 0.683, MK-801 vs. MK-801 + *α*-pinene, p = 0.73).

### 3.5. Effect of *α*-Pinene on MK-801-Induced Abnormal Behaviour in the Hot Plate Test and Neuromuscular Strength Test

**Figure 5:**
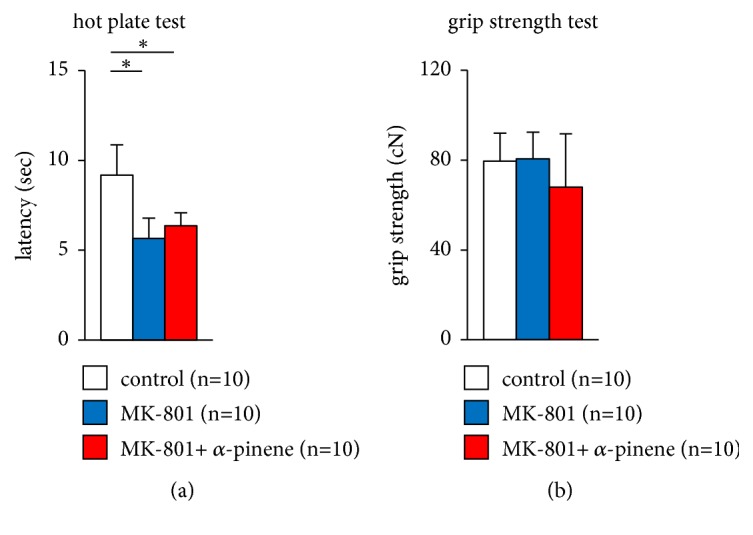
Effect of *α*-pinene on MK-801-induced antinociceptive effects in the hot plate test and grip strength test. (a) Latency to the first fore- or hind-paw response in the hot plate test. (b) Grip strength. All data are presented as box plots. *∗*, significant difference among groups (p < 0.05). The p-values were calculated using one-way analysis of variance in ((a)–(c)).

We examined the effect of *α*-pinene on sensitivity to a painful stimulus in the hot plate test. Administration of MK-801 markedly decreased latency for the mice to lick their front paw (Figure 5(a),* F*_2,28_ = 21.681, p < 0.001; control vs. MK-801, p < 0.001; control vs. MK-801 + *α*-pinene, p < 0.001, MK-801 vs. MK-801 + *α*-pinene, p = 0.4569). All mice administered MK-801 appeared to have a significantly higher pain threshold than mice administered saline (Figure 5(a)). No differences were observed in latency between mice administered MK-801 preexposed to *α*-pinene and exposed to saline.

We compared the neuromuscular strength of mice preexposed to *α*-pinene and mice preexposed to saline administered MK-801. No differences were observed in grip strength among the three groups (Figure 5(b),* F*_2,28_ = 1.622, p = 0.217; control vs. MK-801, p = 0.99; control vs. MK-801 + *α*-pinene, p = 0.306, MK-801 vs. MK-801 + *α*-pinene, p = 0.264).

### 3.6. Effect of *α*-Pinene on MK-801-Induced Abnormal Behaviour in the Tail Suspension Test

**Figure 6:**
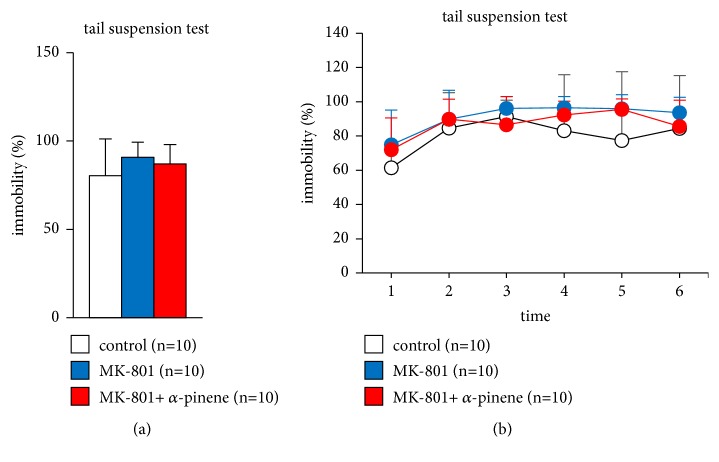
Effect of *α*-pinene on MK-801-induced antidepressant behaviour in the tail suspension test. Graphs showing the proportion of total time spent immobile (a) and the proportion of time spent immobile in each 1-min period (b) in the tail suspension test. All data are presented as box plots. *∗*, significant difference among groups (p < 0.05). The p-values were calculated using one-way analysis of variance in (a) and two-way repeated measures analysis of variance in (b).

We evaluated depressive-like behaviour in mice administered MK-801 after *α*-pinene inhalation. In the tail suspension test, we found no significant differences among the three groups (Figure 6(a),* F*_2,28_ = 1.247, p = 0.304; control vs. MK-801, p = 0.284; control vs. MK-801 + *α*-pinene, p = 0.576, MK-801 vs. MK-801 + *α*-pinene, p = 0.838, B,* F*_2,28_ = 0.109, p = 0.897; control vs. MK-801, p = 0.398; control vs. MK-801 + *α*-pinene, p = 0.965, MK-801 vs. MK-801 + *α*-pinene, p = 1.0).

## 4. Discussion

In this study, we evaluated the ability of *α*-pinene, which is widely used for food flavouring, to suppress and treat abnormal behaviour in a MK-801-induced mental disease mouse model. We investigated whether abnormal behaviour induced by administration of MK-801 would be alleviated by inhalation of *α*-pinene by conducting the open field, Y-maze, tail suspension, and elevated plus maze tests. The main finding of this study was that inhalation of *α*-pinene significantly reduced the abnormal behaviour of mice (hyperactivity and decreased anxiety-like behaviours) induced by MK-801 administration. To our knowledge, this is the first report that inhalation of *α*-pinene can reduce behavioural changes induced by NMDA receptor antagonists. Therefore, our findings indicate that *α*-pinene has potential antipsychotic activity in animal models of schizophrenia.

Open field testing is a useful behavioural experiment that is widely used to evaluate animal spontaneous activity and anxiety-like behaviour [58, 59]. It has been reported that spontaneous activity decreases by intraperitoneal administration of *α*-pinene to rats [60]. However, in the open field test, the amount of activity did not decrease in mice that were preexposed to *α*-pinene. However, during inhalation of *α*-pinene, a decrease in activity was confirmed in the locomotor activity test. Differences in these results are presumed to be due to differences in animal species, methods of administering *α*-pinene, concentration of administration, and inhalation time.

Similar behaviour was observed in this study, as it has been shown that administration of MK-801 in rodents increases activity in open field tests [61, 62]. However, *α*-pinene did not have the effect of suppressing the increase in activity in the open field test and locomotor activity test. In contrast, inhalation of *α*-pinene suppressed the MK-801-induced decrease in anxiety-like behaviour. This may be because the cognitive function of mice was not reduced by the sedative effect of *α*-pinene [63]. Although further research is needed to elucidate the clear mechanism, this experimental result strongly suggests that *α*-pinene reduces abnormal behaviours induced by MK-801.

As described in the introduction section, several studies have already reported the effects of inhalation of *α*-pinene [7, 17, 20, 25]. It has not been reported that inhalation of *α*-pinene suppresses psychiatric disorder-like behaviour. The purpose of this study is to clarify whether MK-801 induced behavioural abnormalities can be suppressed by inhalation of *α*-pinene. Therefore, the group who only inhaled *α*-pinene was excluded.

MK-801 administration is considered to cause an increase in activity level and a decrease in anxiety-like behaviour in the elevated plus maze [64, 65]. Moreover, in this study, administration of MK-801 caused an increase in activity level and a decrease in anxiety-like behaviour in mice. *α*-Pinene did not alleviate behavioural abnormalities in mice induced by administration of MK-801 in this experiment.

The spontaneous alteration score in the Y-maze test is an index of working memory and spatial cognitive function [66, 67]. Administration of MK-801 reportedly reduces spontaneous alteration scores in rodents [68, 69]. In this study, the spontaneous alteration scores of MK-801 treated mice tended to be lower than those of control mice, but no significant difference was found among the three groups. In fact, it has been reported that spontaneous alteration does not decrease significantly by MK-801 administration [70]. Differences in these results are presumed to be due to differences in mouse strain or MK-801 administration concentration. However, in this Y-maze test, MK-801 caused an increase in activity level in mice, while *α*-pinene inhibited the increase in activity level induced by MK-801.

It is known that NMDA receptors are involved in pain associated with peripheral tissues or nerve injuries [71–73]. It has already been reported that administration of MK-801 to mice causes an antinociceptive effect in the hot plate test [74]. The present study also showed that MK-801 has an analgesic effect in acute pain as previously reported. *α*-Pinene did not affect the MK-801-induced analgesic effect. Since the tail flick test and the acetic acid writhing test were not carried out and although details of the influence of *α*-pinene are unknown, *α*-pinene may not affect the peripheral sensory nervous system.

Several basic and clinical studies have shown that the glutamatergic system is widely involved in the pathophysiology of depression [75, 76]. Therefore, administration of the NMDA receptor antagonist MK-801 exerts an antidepressant effect in an animal model of depression [77, 78]. However, in this study, MK-801 administered mice showed no antidepressive behaviour. In this study, we did not use a mouse model of depression under chronic stress as was used in previous reports [77, 78]. The difference in results is presumed to be due to the mouse model used. At least our study result showed that *α*-pinene may not exert antidepressant effects against sudden stress. In fact, in order to investigate the antidepressant effect of *α*-pinene, studies using mouse models of depression are necessary.

It has been reported that inhalation of *α*-pinene causes anxiolytic effects on mice during the elevated plus maze test [25] and antidepressant effects on rats in the forced swim test [79]. However, these results are from daily inhalation. In this experiment, we did not conduct some behavioural experiments using the group that inhaled only *α*-pinene for 30 minutes. Considering previous studies, it is speculated that *α*-pinene inhalation for only 30 minutes does not affect the normal behaviour of mice, but further studies are needed to clarify these observations.

*α*-Pinene promotes the function of the GABA-A receptor and increases the postsynaptic GABA-dependent chloride flow in GABA-A receptors [80]. Furthermore, it has been reported that in mice, after oral ingestion, *α*-pinene binds to the benzodiazepine site of the GABA receptor and becomes a useful hypnotic agent [81]. As reported above, it is presumed that *α*-pinene acts on the brain through stimulation of the GABA receptor [82]. Benzodiazepines such as diazepam also exert sedative and anxiolytic effects via GABA receptors [83]. Conversely, monoterpenes such as *α*-pinene, citronellal, citronellol, and myrcene are reported NMDA receptors antagonists [84]. That is, *α*-pinene is considered to exert physiological effects such as anxiolytic and antioxidant effects through these mechanisms.

Importantly, it is not clear how odour molecules act on nerves through inhalation. One likely hypothesis is that the volatile compound *α*-pinene acts pharmacologically by entering the bloodstream through the mucosa of the nose or lung. The skin permeability of a drug has a high correlation with the lipid solubility or lipophilicity of the drug [85]. Drugs with molecular weights up to 100 kDa are easily absorbed from the nasal mucosa [86]. Since *α*-pinene has a low molecular weight and high lipophilicity [15, 16], it is considered that it is easily absorbed from the nasal mucosa. For the same reasons, it is also expected to have good permeability in brain tissue. In mice, inhaled *α*-pinene has been shown to reach the brain [25]. From these reports, it is reasonable to hypothesise that inhaled *α*-pinene acts on the nerves and alleviates MK-801-induced neural activity abnormality. However, to our knowledge, there are currently no data on the pharmacokinetic interaction between *α*-pinene and MK-801. Further research is needed to clarify these issues.

This study is the first to analyse the effects of *α*-pinene inhalation on MK-801-induced behavioural abnormalities in mice through a series of behavioural tests. Our findings show that the odour of *α*-pinene-containing foodstuffs and essential oils has the effect of alleviating behavioural abnormalities associated with schizophrenia and at the same time presents the scientific basis for the physiological action of odours.

## 5. Conclusions

In conclusion, inhalation of *α*-pinene reduces MK-801-induced psychiatric-like behavioural abnormalities in mice. Our results suggest that inhalation of essential oils containing *α*-pinene acts on nerves and suppresses abnormal activity increase of nerve cells. Thus, *α*-pinene can be a useful natural substance for the treatment and prevention of neuropsychiatric disorders.

## Data Availability

All relevant data are within the article.

## Abbreviations

NMDA: N-methyl-D-aspartate
ANOVA: Analysis of variance.

## References

[1] Pantelis C., Stuart G. W., Nelson H. E., Robbins T. W., Barnes T. R. (2001). Spatial Working Memory Deficits in Schizophrenia: Relationship With Tardive Dyskinesia and Negative Symptoms. *The American Journal of Psychiatry*.

[2] Bora E., Yucel M., Pantelis C. (2009). Cognitive functioning in schizophrenia, schizoaffective disorder and affective psychoses: meta-analytic study. *The British Journal of Psychiatry*.

[3] Keefe R. S. E., Bilder R. M., Davis S. M. (2007). Neurocognitive effects of antipsychotic medications in patients with chronic schizophrenia in the CATIE trial. *Archives of General Psychiatry*.

[4] Gao K., Kemp D. E., Ganocy S. J., Gajwani P., Xia G., Calabrese J. R. (2008). Antipsychotic-induced extrapyramidal side effects in bipolar disorder and schizophrenia: a systematic review. *Journal of Clinical Psychopharmacology*.

[5] Nasrallah H. A., Keshavan M. S., Benes F. M. (2009). Proceedings and data from the Schizophrenia Summit: A critical appraisal to improve the management of schizophrenia. *Journal of Clinical Psychiatry*.

[6] Sakurai H., Bies R. R., Stroup S. T. (2013). Dopamine D2 Receptor Occupancy and Cognition in Schizophrenia: Analysis of the CATIE Data. *Schizophrenia Bulletin*.

[7] Lv X. N., Liu Z. J., Zhang H. J., Tzeng C. M. (2013). Aromatherapy and the central nerve system (CNS): therapeutic mechanism and its associated genes. *Current Drug Targets*.

[8] Zhang Y., Wu Y., Chen T. (2013). Assessing the Metabolic Effects of Aromatherapy in Human Volunteers. *Evidence-Based Complementary and Alternative Medicine*.

[9] Lis-Balchin M. (1997). Essential oils and 'aromatherapy': their modern role in healing. *Journal of the Royal Society of Health*.

[10] De Sousa D. P., De Almeida Soares Hocayen P., Andrade L. N., Andreatini R. A. (2015). A systematic review of the anxiolytic-like effects of essential oils in animal models. *Molecules*.

[11] Simonsen J., Ross W. C. (1957). *The Triterpenes and Their Derivative S Hydroxy Acids, Hydroxy Lactones, Hydroxyaldehydo a Hardcover*.

[12] Pereira Limberger R., Mendes Aleixo A., Fett-Neto A. G., T. Henriques A. (2007). Bioconversion of (+)- and (-)-alpha-pinene to (+)- and (-)-verbenone by plant cell cultures of Psychotria brachyceras and Rauvolfia sellowii. *Electronic Journal of Biotechnology*.

[13] Da Silva A. C. R., Lopes P. M., De Azevedo M. M. B., Costa D. C. M., Alviano C. S., Alviano D. S. (2012). Biological activities of *α*-pinene and *β*-pinene enantiomers. *Molecules*.

[14] U.S. Food and Drug Administration.

[15] Falk A., Gullstrand E., Wigaeus-Hjelm E. (1990). Liquid/air partition coefficients of four terpenes. *British Journal of Industrial Medicine*.

[16] Falk A. A., Hagberg M. T., Lof A. E., Wigaeus-Hjelm E. M., Wang Z. P. (1990). Uptake, distribution and elimination of alpha-pinene in man after exposure by inhalation.. *Scandinavian Journal of Work, Environment & Health*.

[17] Mercier B., Prost J., Prost M. (2009). The essential oil of turpentine and its major volatile fraction (*α*- and *β*-pinenes): A review. *International Journal of Occupational Medicine and Environmental Health*.

[18] Martin S., Padilla E., Ocete M., Galvez J., Jiménez J., Zarzuelo A. (1993). Anti-Inflammatory Activity of the Essential Oil of *Bupleurum fruticescens*. *Planta Medica*.

[19] Zhou J. Y., Tang F. D., Mao G. G., Bian R. L. (2004). Effect of alpha-pinene on nuclear translocation of NF-kappa B in THP-1 cells. *Acta Pharmacologica Sinica*.

[20] Ahmad A., Husain A., Mujeeb M. (2013). A review on therapeutic potential of *Nigella sativa*: a miracle herb. *Asian Pacific Journal of Tropical Biomedicine*.

[21] Consroe P., Martin A., Singh V. (1981). Antiepileptic potential of cannabidiol analogs. *The Journal of Clinical Pharmacology*.

[22] Singh H. P., Batish D. R., Kaur S., Arora K., Kohli R. K. (2006). *α*-Pinene inhibits growth and induces oxidative stress in roots. *Annals of Botany*.

[23] Eftekhar F., Yousefzadi M., Borhani K. (2004). Antibacterial activity of the essential oil from Ferula gummosa seed. *Fitoterapia*.

[24] Him A., Ozbek H., Turel I., Oner A. C. (2008). Antinociceptive activity of alpha-pinene and fenchone. *Pharmacologyonline*.

[25] Satou T., Kasuya H., Maeda K., Koike K. (2014). Daily Inhalation of *α*-Pinene in Mice: Effects on Behavior and Organ Accumulation. *Phytotherapy Research*.

[26] Yamaoka S. (2005). Effects of Plant-derived Odors on Sleep-Wakefulness and Circadian Rhythmicity in Rats. *Chemical Senses*.

[27] Okamura N., Hashimoto K., Shimizu E., Kumakiri C., Komatsu N., Iyo M. (2004). Adenosine A1 Receptor Agonists Block the Neuropathological Changes in Rat Retrosplenial Cortex after Administration of the NMDA Receptor Antagonist Dizocilpine. *Neuropsychopharmacology*.

[28] Xi D., Zhang W., Wang H., Stradtman G. G., Gao W. (2009). Dizocilpine (MK-801) induces distinct changes of N-methyl-d-aspartic acid receptor subunits in parvalbumin-containing interneurons in young adult rat prefrontal cortex. *The International Journal of Neuropsychopharmacology*.

[29] Svoboda J., Stankova A., Entlerova M., Stuchlik A. (2015). Acute administration of MK-801 in an animal model of psychosis in rats interferes with cognitively demanding forms of behavioral flexibility on a rotating arena. *Frontiers in Behavioral Neuroscience*.

[30] Murueta-Goyena A. L., Odrioizola A. B., Gargiulo P. A., Sánchez J. V. L. (2017). Neuropathological background of mk-801 for inducing murine model of schizophrenia. *Psychiatry and Neuroscience Update*.

[31] Behrens M. M., Ali S. S., Dao D. N. (2007). Ketamine-induced loss of phenotype of fast-spiking interneurons is mediated by NADPH-oxidase. *Science*.

[32] Braun I., Genius J., Grunze H., Bender A., Möller H., Rujescu D. (2007). Alterations of hippocampal and prefrontal GABAergic interneurons in an animal model of psychosis induced by NMDA receptor antagonism. *Schizophrenia Research*.

[33] Gilmour G., Dix S., Fellini L. (2012). NMDA receptors, cognition and schizophrenia—testing the validity of the NMDA receptor hypofunction hypothesis. *Neuropharmacology*.

[34] Jadi M. P., Behrens M. M., Sejnowski T. J. (2016). Abnormal Gamma Oscillations in N-Methyl-D-Aspartate Receptor Hypofunction Models of Schizophrenia. *Biological Psychiatry*.

[35] Dauvermann M. R., Lee G., Dawson N. (2017). Glutamatergic regulation of cognition and functional brain connectivity: insights from pharmacological, genetic and translational schizophrenia research. *British Journal of Pharmacology*.

[36] Javitt D. C. (2004). Glutamate as a therapeutic target in psychiatric disorders. *Molecular Psychiatry*.

[37] Hashimoto K., Okamura N., Shimizu E., Iyo M. (2004). Glutamate Hypothesis of Schizophrenia and Approach for Possible Therapeutic Drugs. *Current Medicinal Chemistry-Central Nervous System Agents*.

[38] Coyle J. T. (2006). Substance use disorders and schizophrenia: A question of shared glutamatergic mechanisms. *Neurotoxicity Research*.

[39] Conn P. J., Tamminga C., Schoepp D. D., Lindsley C. (2008). Schizophrenia: Moving Beyond Monoamine Antagonists. *Molecular Interventions*.

[40] Lisman J. E., Coyle J. T., Green R. W. (2008). Circuit-based framework for understanding neurotransmitter and risk gene interactions in schizophrenia. *Trends in Neurosciences*.

[41] Jeon S. J., Kim E., Lee J. S. (2017). Maslinic acid ameliorates NMDA receptor blockade-induced schizophrenia-like behaviors in mice. *Neuropharmacology*.

[42] Enomoto T., Ishibashi T., Tokuda K., Ishiyama T., Toma S., Ito A. (2008). Lurasidone reverses MK-801-induced impairment of learning and memory in the Morris water maze and radial-arm maze tests in rats. *Behavioural Brain Research*.

[43] Bubser M., Bridges T. M., Dencker D. (2014). Selective Activation of M 4 Muscarinic Acetylcholine Receptors Reverses MK-801-Induced Behavioral Impairments and Enhances Associative Learning in Rodents. *ACS Chemical Neuroscience*.

[44] Park S. J., Lee Y., Oh H. K. (2014). Oleanolic acid attenuates MK-801-induced schizophrenia-like behaviors in mice. *Neuropharmacology*.

[45] O'Neill M. F., Hicks C. A., Shaw G., Parameswaran T., Cardwell G. P., O'Neill M. J. (1998). Effects of 5-hydroxytryptamine2 receptor antagonism on the behavioral activation and immediate early gene expression induced by dizocilpine. *The Journal of Pharmacology and Experimental Therapeutics*.

[46] Gray L., van den Buuse M., Scarr E., Dean B., Hannan A. J. (2009). Clozapine reverses schizophrenia-related behaviours in the metabotropic glutamate receptor 5 knockout mouse: association with N-methyl-d-aspartic acid receptor up-regulation. *The International Journal of Neuropsychopharmacology*.

[47] Bygrave A. M., Masiulis S., Nicholson E. (2016). Knockout of NMDA-receptors from parvalbumin interneurons sensitizes to schizophrenia-related deficits induced by MK-801. *Translational Psychiatry*.

[48] Ueno H., Shimada A., Suemitsu S. (2019). Anti-depressive-like effect of 2-phenylethanol inhalation in mice. *Biomedicine & Pharmacotherapy*.

[49] Koshimizu H., Takao K., Matozaki T., Ohnishi H., Miyakawa T., Tang Y. (2014). Comprehensive Behavioral Analysis of Cluster of Differentiation 47 Knockout Mice. *PLoS ONE*.

[50] Rompala G. R., Zsiros V., Zhang S., Kolata S. M., Nakazawa K., Yoshikawa T. (2013). Contribution of NMDA Receptor Hypofunction in Prefrontal and Cortical Excitatory Neurons to Schizophrenia-Like Phenotypes. *PLoS ONE*.

[51] Matsuda I., Shoji H., Yamasaki N., Miyakawa T., Aiba A. (2016). Comprehensive behavioral phenotyping of a new Semaphorin 3 F mutant mouse. *Molecular Brain*.

[52] Bansal P. K., Deshmukh R. (2017). *Animal Models of Neurological Disorders. Principle and Working Procedure for Animal Models of Neurological Disorders*.

[53] Okonogi T., Nakayama R., Sasaki T., Ikegaya Y. (2018). Characterization of peripheral activity states and cortical local field potentials of mice in an elevated plus maze test. *Frontiers in Behavioral Neuroscience*.

[54] Wolf A., Bauer B., Abner E. L., Ashkenazy-Frolinger T., Hartz A. M. S. (2016). A comprehensive behavioral test battery to assess learning and memory in 129S6/Tg2576 mice. *PLoS ONE*.

[55] Vogel H. (2007). *Drug Discovery and Evaluation: Pharmacological Assays*.

[56] Nakatani J., Tamada K., Hatanaka F. (2009). Abnormal behavior in a chromosome-engineered mouse model for human 15q11-13 duplication seen in autism. *Cell*.

[57] Chartoff E. H., Heusner C. L., Palmiter R. D. (2005). Dopamine is not Required for the Hyperlocomotor Response to NMDA Receptor Antagonists. *Neuropsychopharmacology*.

[58] Belzung C., Crusio W. E., Gerlai R. T. (1999). Measuring exploratory behavior. *Handbook of molecular genetics techniques for brain and behavior research (techniques in the behavioral and neural sciences)*.

[59] Prut L., Belzung C. (2003). The open field as a paradigm to measure the effects of drugs on anxiety-like behaviors: a review. *European Journal of Pharmacology*.

[60] Zamyad M., Abasnejad M., Esmaeili-Mahani S., Mostafavi A. (2016). Alpha-Pinene as the Main Component of Ducrosia anethifolia (Boiss) Essential Oil is Responsible for its Effect on Locomotor Activity in Rats. *Avicenna Journal of Neuro Psych Physiology*.

[61] Dai H., Carey R. J. (1994). The NMDA antagonist MK-801 can impair attention to exteroceptive stimuli. *Behavioural Brain Research*.

[62] Plaznik A., Palejko W., Nazar M., Jessa M. (1994). Effects of antagonists at the NMDA receptor complex in two models of anxiety. *European Neuropsychopharmacology*.

[63] Lee G. Y., Lee C., Park G. H., Jang J. H. (2017). Amelioration of Scopolamine-Induced Learning and Memory Impairment by *α*-Pinene in C57BL/6 Mice. *Evidence-Based Complementary and Alternative Medicine*.

[64] Sharma A. C., Kulkarni S. K. (1991). MK-801 produces antianxiety effect in elevated plus-maze in mice. *Drug Development Research*.

[65] Bertoglio L. J., Carobrez A. P. (2003). Anxiolytic-like effects of NMDA/glycine-B receptor ligands are abolished during the elevated plus-maze trial 2 in rats. *Psychopharmacology*.

[66] Choy K. H. C., de Visser Y., Nichols N. R., van den Buuse M. (2008). Combined neonatal stress and young-adult glucocorticoid stimulation in rats reduce BDNF expression in hippocampus: effects on learning and memory. *Hippocampus*.

[67] Shipton O. A., El-Gaby M., Apergis-Schoute J. (2014). Left–right dissociation of hippocampal memory processes in mice. *Proceedings of the National Acadamy of Sciences of the United States of America*.

[68] Mihara T., Mihara K., Yarimizu J. (2007). Pharmacological Characterization of a Novel, Potent Adenosine A1 and A2A Receptor Dual Antagonist, 5-[5-Amino-3-(4-fluorophenyl)pyrazin-2-yl]-1-isopropylpyridine-2(1H)-one (ASP5854), in Models of Parkinson's Disease and Cognition. *The Journal of Pharmacology and Experimental Therapeutics*.

[69] Okuyama S., Fukata T., Nishigawa Y. (2013). Citrus flavonoid improves MK-801-induced locomotive hyperactivity: Possible relevance to schizophrenia. *Journal of Functional Foods*.

[70] Hasegawa Y., Inoue T., Kawaminami S., Fujita M. (2016). Effects of scallop shell extract on scopolamine-induced memory impairment and MK801-induced locomotor activity. *Asian Pacific Journal of Tropical Medicine*.

[71] Petrenko A. B., Yamakura T., Baba H., Shimoji K. (2003). The Role of N-Methyl-d-Aspartate (NMDA) Receptors in Pain: A Review. *Anesthesia & Analgesia*.

[72] Willert R. P., Woolf C. J., Hobson A. R., Delaney C., Thompson D. G., Aziz Q. (2004). The Development and Maintenance of Human Visceral Pain Hypersensitivity Is Dependent on the N-Methyl-D-Aspartate Receptor. *Gastroenterology*.

[73] McRoberts J., Ennes H., Marvizón J., Fanselow M., Mayer E., Vissel B. (2011). Selective knockdown of NMDA receptors in primary afferent neurons decreases pain during phase 2 of the formalin test. *Neuroscience*.

[74] Meymandi M. S., Keyhanfar F., Sepehri G. R., Heravi G., Yazdanpanah O. (2017). The contribution of NMDA receptors in antinociceptive effect of pregabalin: Comparison of two models of pain assessment. *Anesthesiology and Pain Medicine*.

[75] Paul I. A., Skolnick P. (2003). Glutamate and Depression. *Annals of the New York Academy of Sciences*.

[76] Mitani H., Shirayama Y., Yamada T., Maeda K., Ashby C. R., Kawahara R. (2006). Correlation between plasma levels of glutamate, alanine and serine with severity of depression. *Progress in Neuro-Psychopharmacology & Biological Psychiatry*.

[77] Mantovani M., Pértile R., Calixto J. B., Santos A. R. S., Rodrigues A. L. S. (2003). Melatonin exerts an antidepressant-like effect in the tail suspension test in mice: evidence for involvement of N-methyl-D-aspartate receptors and the L-arginine-nitric oxide pathway. *Neuroscience Letters*.

[78] Sanacora G., Zarate C. A., Krystal J. H., Manji H. K. (2008). Targeting the glutamatergic system to develop novel, improved therapeutics for mood disorders. *Nature Reviews Drug Discovery*.

[79] Kong Y., Wang T., Wang R. (2017). Inhalation of Roman chamomile essential oil attenuates depressive-like behaviors in Wistar Kyoto rats. *Science China Life Sciences*.

[80] Aoshima H., Hamamoto K. (1999). Potentiation of GABAA receptors expressed in Xenopus oocytes by perfume and phytoncid. *Bioscience, Biotechnology, and Biochemistry*.

[81] Yang H., Woo J., Pae A. N. (2016). *α*-pinene, a major constituent of pine tree oils, enhances non-rapid eye movement sleep in mice through GABAA-benzodiazepine receptors. *Molecular Pharmacology*.

[82] Kasture V. S., Chopde C. T., Deshmukh V. K. (2000). Anticonvulsive activity of Albizzia lebbeck, Hibiscus rosa sinesis and Butea monosperma in experimental animals. *Journal of Ethnopharmacology*.

[83] Brunton L., Lazo J., Parker K. (2006). *Goodman And Gilman's The Pharmacological Basis of Therapeutics*.

[84] Guimarães A. G., Quintans J. S. S., Quintans-Júnior L. J. (2013). Monoterpenes with analgesic activity—a systematic review. *Phytotherapy Research*.

[85] Scheuplein R., Blank I., Brauner G., Macfarlane D. J. (1969). Percutaneous Absorption of Steroids∗. *Journal of Investigative Dermatology*.

[86] Fisher A. N., Brown K., Davis S. S., Parr G. D., Smith D. A. (1987). The effect of molecular size on the nasal absorption of water‐soluble compounds in the albino rat. *Journal of Pharmacy and Pharmacology*.

